# 1-Benz­yloxy-2,5-bis­(chloro­meth­yl)-4-meth­oxy­benzene

**DOI:** 10.1107/S1600536812029558

**Published:** 2012-07-04

**Authors:** Hager Trad, Mustapha Majdoub, Mohamed Salah Belkhiria

**Affiliations:** a University of Monastir, Faculté de Pharmacie de Monastir, Avenue Avicenne, 5019 Monastir, Tunisia; b University of Monastir, Faculté des Sciences de Monastir, Avenue de l’Environnement, 5019 Monastir, Tunisia

## Abstract

In the title compound, C_16_H_16_Cl_2_O_2_, the dihedral angle between the two rings is 52.65 (10)°. The two Cl atoms are *trans* to one another being displaced by 1.644 (5) and −1.664 (4) Å from the plane of the benzene ring. Except for the two Cl atoms and the C atoms of the ring of the benz­yloxy group, all the other atoms of the compound lie in the same plane [maximum deviation = 0.056 (3) Å]. In the crystal, no significant intermolecular interactions are observed.

## Related literature

For general background, physical properties and synthesis of poly(*p*-phenyl­ene­vinyl­ene) derivatives (PPVs), see: Trad *et al.* (2006 ▶). For related structures, see: Huang *et al.* (2011 ▶); Watanabe *et al.* (2005 ▶).
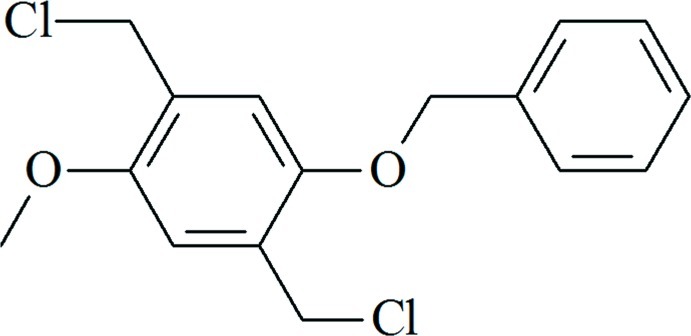


## Experimental

### 

#### Crystal data

C_16_H_16_Cl_2_O_2_
*M* *_r_* = 311.19
Monoclinic, 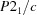

*a* = 10.9026 (4) Å
*b* = 17.8127 (6) Å
*c* = 8.4221 (2) Å
β = 109.561 (4)°
*V* = 1541.21 (9) Å^3^
*Z* = 4
Mo *K*α radiation
μ = 0.42 mm^−1^
*T* = 298 K
0.40 × 0.30 × 0.20 mm

#### Data collection

Enraf–Nonius κ-geometry TurboCAD-4 diffractometer
4141 measured reflections
3363 independent reflections
1562 reflections with *I* > 2σ(*I*)
*R*_int_ = 0.099
1 standard reflections every 60 min intensity decay: 3%

#### Refinement

*R*[*F* ^2^ > 2σ(*F* ^2^)] = 0.049
*wR*(*F* ^2^) = 0.166
*S* = 1.00
3363 reflections
182 parameters
H-atom parameters constrained
Δρ_max_ = 0.26 e Å^−3^
Δρ_min_ = −0.34 e Å^−3^

### 

Data collection: *CAD-4 EXPRESS* (Enraf–Nonius, 1994 ▶); cell refinement: *CAD-4 EXPRESS*; data reduction: *XCAD4* (Harms & Wocadlo, 1995 ▶); program(s) used to solve structure: *SIR2004* (Burla *et al.*, 2005 ▶); program(s) used to refine structure: *SHELXL97* (Sheldrick, 2008 ▶); molecular graphics: *ORTEP-3* (Farrugia, 1997 ▶); software used to prepare material for publication: *SHELXL97*.

## Supplementary Material

Crystal structure: contains datablock(s) I, global. DOI: 10.1107/S1600536812029558/ng5278sup1.cif

Structure factors: contains datablock(s) I. DOI: 10.1107/S1600536812029558/ng5278Isup2.hkl

Supplementary material file. DOI: 10.1107/S1600536812029558/ng5278Isup3.cml

Additional supplementary materials:  crystallographic information; 3D view; checkCIF report

## Figures and Tables

**Fig. 1.**
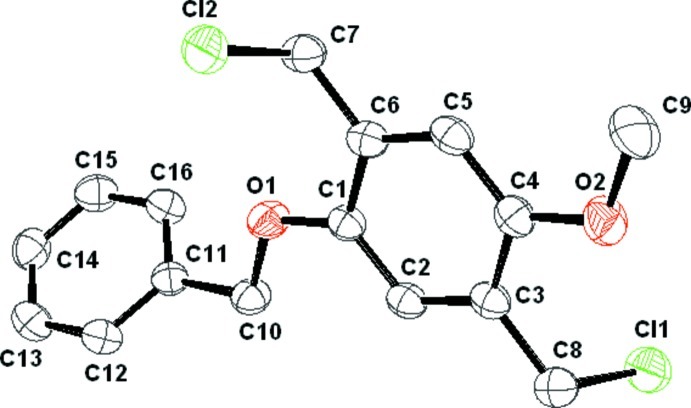
The molecular structure of the title compoud MBzCl with displacement ellipsoids drawn at the 30% probability.

**Fig. 2.**
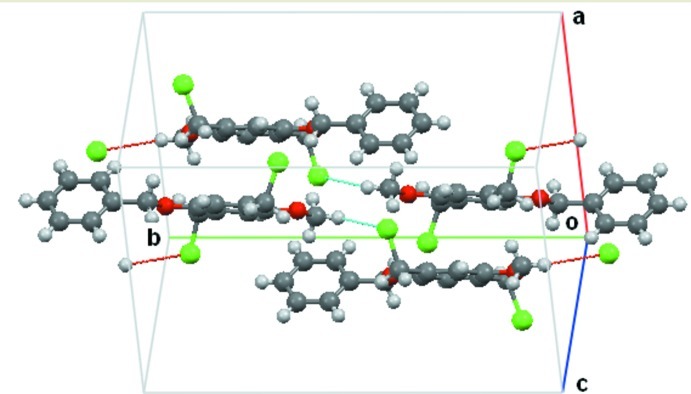
The lattice framework of the title compound MBzCl, showing molecules linked through weak intermolecular C—H···Cl hydrogen bonds to form chains which propagate along [010].

## supplementary crystallographic information

### Comment

The 1-benzyloxy-2,5-bis(chloromethyl)-4-methoxybenzene (MBzCl) was used as a monomer for the synthesis of π-conjugated polymers such as poly(*p*-phenylenevinylene) derivatives (PPVs) which have potential application as electroluminescent materials. The monomer (MBzCl) has been synthesized as described in literature (Trad *et al.*, 2006).

The asymmetric unit of the title compound (MBzCl) contains one molecule which is presented in Fig.1. The dihedral angle between the two phenyl rings is 52.65 (10)°. The two planes containing respectively the two chloromethyl groups and the substituted benzene ring are nearly orthogonal to each other, with a dihedral angle equal to 87.69 (9)°. The two chlorine atoms are in *trans* position with respect to the benzene substituted group. All atoms of the compound (MBzCl) lie in the same plane, the largest deviation being 0.0563 (28) Å for atom C9, except the two chlorine atoms and the carbons of the phenyl of the benzyloxy group. Some selected bond lengths are given in table 2 and agree with those reported for similar compounds (Huang *et al.*, 2011; Watanabe *et al.*, 2005). A strong intramolecular hydrogen bond C7—H7A···O1 is observed (table 1). In the crystal structure, weak intermolecular C—H···Cl hydrogen bonds link molecules of (MBzCl) into chains which propagate along [010] as shown in Fig. 2.

### Experimental

The compound 1-benzyloxy-2,5-bis(chloromethyl)-4-methoxybenzene (MBzCl) was prepared in two steps: a mixture of 4-methoxyphenol (10 mmol), benzylchloride (15 mmol) and K_2_CO_3_ (20 mmol) was added to 10 ml of DMF and was heated under stirring at 353 K for 24 h. The product, 1-benzyloxy-4-methoxybenzene (MBz), was purified by recrystallization from ethanol and was obtained as white powder.Yield: 95%; mp 346 (2) K. In a next step, a suspension of (MBz) (10 mmol) and paraformaldehyde (50 mmol), in a mixture of glacial acetic acid (20 ml) and 37% hydrochloric acid (10 ml), was left to stir for approximately 20 h at room temperature. The resulting mixture was then poured into distilled water. The product (MBzCl) was extracted with dichloromethane and recrystallized from ethanol as colorless needle-like white crystals. Yield: 40%; mp: 388 (2) K.

### Refinement

All H atoms were refined using a riding model with C—H = 0.96 (CH_3_), 0.97 (CH_2_), 0.93 (C_Ar_H) Å and *U_iso_*(H) = 1.5 *U_eq_*(C), 1.2 *U_eq_*(C) and 1.2 *U_eq_*(C) respectively.

### Crystal data

**Table:**

C_16_H_16_Cl_2_O_2_	*F*(000) = 648
*M**_r_* = 311.19	*D*_x_ = 1.341 Mg m^−^^3^
Monoclinic, *P*2_1_/*c*	Mo *K*α radiation, λ = 0.71073 Å
Hall symbol: -P 2ybc	Cell parameters from 4284 reflections
*a* = 10.9026 (4) Å	θ = 2.0–27.0°
*b* = 17.8127 (6) Å	µ = 0.42 mm^−^^1^
*c* = 8.4221 (2) Å	*T* = 298 K
β = 109.561 (4)°	Needle-shaped, colourless
*V* = 1541.21 (9) Å^3^	0.40 × 0.30 × 0.20 mm
*Z* = 4	

### Data collection

**Table:**

Enraf–Nonius κ-geometry TurboCAD-4 diffractometer	*R*_int_ = 0.099
Radiation source: fine-focus sealed tube	θ_max_ = 27.0°, θ_min_ = 2.0°
Graphite monochromator	*h* = −13→1
non–profiled ω/2θ scans	*k* = −22→0
4141 measured reflections	*l* = −10→10
3363 independent reflections	1 standard reflections every 60 min
1562 reflections with *I* > 2σ(*I*)	intensity decay: 3%

### Refinement

**Table:**

Refinement on *F*^2^	Primary atom site location: structure-invariant direct methods
Least-squares matrix: full	Secondary atom site location: difference Fourier map
*R*[*F*^2^ > 2σ(*F*^2^)] = 0.049	Hydrogen site location: inferred from neighbouring sites
*wR*(*F*^2^) = 0.166	H-atom parameters constrained
*S* = 1.00	*w* = 1/[σ^2^(*F*_o_^2^) + (0.0682*P*)^2^ + 0.2353*P*] where *P* = (*F*_o_^2^ + 2*F*_c_^2^)/3
3363 reflections	(Δ/σ)_max_ < 0.001
182 parameters	Δρ_max_ = 0.26 e Å^−^^3^
0 restraints	Δρ_min_ = −0.34 e Å^−^^3^

### Special details

**Table:**

Geometry. All s.u.'s (except the s.u. in the dihedral angle between two l.s. planes) are estimated using the full covariance matrix. The cell s.u.'s are taken into account individually in the estimation of s.u.'s in distances, angles and torsion angles; correlations between s.u.'s in cell parameters are only used when they are defined by crystal symmetry. An approximate (isotropic) treatment of cell s.u.'s is used for estimating s.u.'s involving l.s. planes.
Refinement. Refinement of *F*^2^ against ALL reflections. The weighted *R*-factor *wR* and goodness of fit *S* are based on *F*^2^, conventional *R*-factors *R* are based on *F*, with *F* set to zero for negative *F*^2^. The threshold expression of *F*^2^ > 2σ(*F*^2^) is used only for calculating *R*-factors(gt) *etc*. and is not relevant to the choice of reflections for refinement. *R*-factors based on *F*^2^ are statistically about twice as large as those based on *F*, and *R*- factors based on ALL data will be even larger.

### Fractional atomic coordinates and isotropic or equivalent isotropic displacement parameters (Å^2^)

**Table:**

	*x*	*y*	*z*	*U*_iso_*/*U*_eq_	
Cl1	0.01947 (9)	0.37837 (6)	0.06080 (12)	0.0810 (3)	
Cl2	0.62734 (9)	0.11217 (6)	0.35509 (13)	0.0854 (4)	
O1	0.2844 (2)	0.08686 (11)	0.1620 (3)	0.0591 (6)	
O2	0.3519 (2)	0.39330 (11)	0.2330 (3)	0.0676 (7)	
C1	0.2962 (3)	0.16329 (16)	0.1757 (4)	0.0456 (7)	
C2	0.2106 (3)	0.21379 (16)	0.0689 (4)	0.0488 (7)	
H2	0.1388	0.1960	−0.0179	0.059*	
C3	0.2311 (3)	0.29076 (16)	0.0903 (4)	0.0479 (7)	
C4	0.3386 (3)	0.31709 (16)	0.2217 (4)	0.0510 (7)	
C5	0.4231 (3)	0.26696 (17)	0.3285 (4)	0.0521 (7)	
H5	0.4947	0.2849	0.4155	0.062*	
C6	0.4029 (3)	0.18995 (16)	0.3084 (4)	0.0485 (7)	
C7	0.4947 (3)	0.13664 (18)	0.4269 (4)	0.0596 (8)	
H7A	0.4481	0.0915	0.4368	0.071*	
H7B	0.5289	0.1595	0.5376	0.071*	
C8	0.1388 (3)	0.34438 (18)	−0.0267 (4)	0.0590 (8)	
H8A	0.0950	0.3195	−0.1330	0.071*	
H8B	0.1872	0.3865	−0.0487	0.071*	
C9	0.4639 (4)	0.4225 (2)	0.3585 (5)	0.0789 (11)	
H9A	0.4628	0.4083	0.4679	0.118*	
H9B	0.4640	0.4763	0.3503	0.118*	
H9C	0.5408	0.4028	0.3421	0.118*	
C10	0.1737 (3)	0.05788 (17)	0.0327 (4)	0.0555 (8)	
H10A	0.1737	0.0752	−0.0765	0.067*	
H10B	0.0946	0.0755	0.0491	0.067*	
C11	0.1787 (3)	−0.02635 (16)	0.0394 (4)	0.0480 (7)	
C12	0.1706 (3)	−0.06681 (18)	−0.1013 (4)	0.0599 (8)	
H12	0.1642	−0.0419	−0.2009	0.072*	
C13	0.1719 (4)	−0.14467 (19)	−0.0975 (5)	0.0704 (10)	
H13	0.1663	−0.1717	−0.1941	0.084*	
C14	0.1815 (3)	−0.18163 (19)	0.0491 (5)	0.0683 (9)	
H14	0.1828	−0.2338	0.0522	0.082*	
C15	0.1892 (3)	−0.14167 (19)	0.1900 (5)	0.0678 (9)	
H15	0.1955	−0.1665	0.2896	0.081*	
C16	0.1875 (3)	−0.06377 (17)	0.1849 (4)	0.0596 (8)	
H16	0.1924	−0.0367	0.2813	0.071*	

### Atomic displacement parameters (Å^2^)

**Table:**

	*U*^11^	*U*^22^	*U*^33^	*U*^12^	*U*^13^	*U*^23^
Cl1	0.0677 (6)	0.0993 (7)	0.0726 (6)	0.0214 (5)	0.0192 (5)	−0.0109 (5)
Cl2	0.0632 (6)	0.1105 (8)	0.0773 (7)	0.0212 (5)	0.0167 (5)	−0.0182 (5)
O1	0.0492 (12)	0.0531 (12)	0.0632 (14)	−0.0077 (10)	0.0031 (10)	−0.0008 (10)
O2	0.0667 (15)	0.0520 (13)	0.0768 (17)	−0.0099 (11)	0.0145 (12)	−0.0128 (11)
C1	0.0400 (15)	0.0517 (17)	0.0442 (15)	−0.0052 (12)	0.0131 (12)	−0.0040 (13)
C2	0.0415 (16)	0.0598 (19)	0.0440 (16)	−0.0063 (13)	0.0128 (13)	−0.0056 (14)
C3	0.0436 (15)	0.0592 (18)	0.0431 (16)	−0.0039 (14)	0.0174 (13)	−0.0044 (13)
C4	0.0501 (17)	0.0543 (18)	0.0517 (17)	−0.0056 (14)	0.0212 (14)	−0.0068 (14)
C5	0.0444 (16)	0.0630 (19)	0.0467 (16)	−0.0106 (14)	0.0124 (13)	−0.0118 (14)
C6	0.0389 (14)	0.0627 (18)	0.0444 (15)	−0.0050 (13)	0.0147 (12)	−0.0015 (14)
C7	0.0509 (18)	0.072 (2)	0.0514 (18)	0.0006 (15)	0.0107 (15)	0.0008 (15)
C8	0.0568 (19)	0.065 (2)	0.0530 (19)	0.0010 (16)	0.0149 (15)	0.0020 (16)
C9	0.089 (3)	0.059 (2)	0.080 (3)	−0.0217 (19)	0.016 (2)	−0.0175 (18)
C10	0.0468 (16)	0.0556 (19)	0.0553 (19)	−0.0050 (14)	0.0054 (14)	−0.0018 (14)
C11	0.0381 (14)	0.0526 (17)	0.0493 (17)	−0.0051 (13)	0.0091 (12)	−0.0032 (14)
C12	0.063 (2)	0.066 (2)	0.0474 (19)	−0.0032 (16)	0.0135 (15)	−0.0014 (15)
C13	0.077 (2)	0.069 (2)	0.061 (2)	−0.0076 (19)	0.0188 (19)	−0.0162 (18)
C14	0.068 (2)	0.0512 (19)	0.082 (3)	−0.0034 (16)	0.0195 (19)	−0.0040 (19)
C15	0.070 (2)	0.068 (2)	0.062 (2)	−0.0075 (17)	0.0186 (17)	0.0099 (17)
C16	0.064 (2)	0.062 (2)	0.0509 (19)	−0.0076 (16)	0.0163 (15)	−0.0045 (15)

### Geometric parameters (Å, º)

**Table:**

Cl1—C8	1.801 (3)	C8—H8B	0.9700
Cl2—C7	1.798 (3)	C9—H9A	0.9600
O1—C1	1.369 (3)	C9—H9B	0.9600
O1—C10	1.424 (3)	C9—H9C	0.9600
O2—C4	1.365 (3)	C10—C11	1.502 (4)
O2—C9	1.420 (4)	C10—H10A	0.9700
C1—C2	1.388 (4)	C10—H10B	0.9700
C1—C6	1.399 (4)	C11—C12	1.364 (4)
C2—C3	1.391 (4)	C11—C16	1.370 (4)
C2—H2	0.9300	C12—C13	1.387 (4)
C3—C4	1.397 (4)	C12—H12	0.9300
C3—C8	1.494 (4)	C13—C14	1.372 (5)
C4—C5	1.378 (4)	C13—H13	0.9300
C5—C6	1.391 (4)	C14—C15	1.363 (5)
C5—H5	0.9300	C14—H14	0.9300
C6—C7	1.493 (4)	C15—C16	1.388 (4)
C7—H7A	0.9700	C15—H15	0.9300
C7—H7B	0.9700	C16—H16	0.9300
C8—H8A	0.9700		
			
C1—O1—C10	117.2 (2)	H8A—C8—H8B	108.0
C4—O2—C9	117.4 (3)	O2—C9—H9A	109.5
O1—C1—C2	124.5 (2)	O2—C9—H9B	109.5
O1—C1—C6	115.8 (3)	H9A—C9—H9B	109.5
C2—C1—C6	119.8 (3)	O2—C9—H9C	109.5
C1—C2—C3	120.7 (3)	H9A—C9—H9C	109.5
C1—C2—H2	119.6	H9B—C9—H9C	109.5
C3—C2—H2	119.6	O1—C10—C11	108.8 (2)
C2—C3—C4	119.3 (3)	O1—C10—H10A	109.9
C2—C3—C8	120.1 (3)	C11—C10—H10A	109.9
C4—C3—C8	120.6 (3)	O1—C10—H10B	109.9
O2—C4—C5	124.5 (3)	C11—C10—H10B	109.9
O2—C4—C3	115.5 (3)	H10A—C10—H10B	108.3
C5—C4—C3	120.0 (3)	C12—C11—C16	119.0 (3)
C4—C5—C6	121.1 (3)	C12—C11—C10	120.3 (3)
C4—C5—H5	119.5	C16—C11—C10	120.7 (3)
C6—C5—H5	119.5	C11—C12—C13	120.7 (3)
C5—C6—C1	119.2 (3)	C11—C12—H12	119.7
C5—C6—C7	120.2 (3)	C13—C12—H12	119.7
C1—C6—C7	120.7 (3)	C14—C13—C12	119.9 (3)
C6—C7—Cl2	111.3 (2)	C14—C13—H13	120.1
C6—C7—H7A	109.4	C12—C13—H13	120.1
Cl2—C7—H7A	109.4	C15—C14—C13	119.8 (3)
C6—C7—H7B	109.4	C15—C14—H14	120.1
Cl2—C7—H7B	109.4	C13—C14—H14	120.1
H7A—C7—H7B	108.0	C14—C15—C16	119.9 (3)
C3—C8—Cl1	111.4 (2)	C14—C15—H15	120.0
C3—C8—H8A	109.4	C16—C15—H15	120.0
Cl1—C8—H8A	109.4	C11—C16—C15	120.7 (3)
C3—C8—H8B	109.4	C11—C16—H16	119.7
Cl1—C8—H8B	109.4	C15—C16—H16	119.7

### Hydrogen-bond geometry (Å, º)

**Table:**

*D*—H···*A*	*D*—H	H···*A*	*D*···*A*	*D*—H···*A*
C7—H7*A*···O1	0.97	2.40	2.756 (4)	101
